# X-ray Pulsar Signal Denoising Based on Variational Mode Decomposition

**DOI:** 10.3390/e23091181

**Published:** 2021-09-08

**Authors:** Qiang Chen, Yong Zhao, Lixia Yan

**Affiliations:** School of Automation Science and Electrical Engineering, Beihang University (BUAA), Beijing 100191, China; qiangchen@buaa.edu.cn (Q.C.); zhaoyong1996@buaa.edu.cn (Y.Z.)

**Keywords:** X-ray pulsar, signal denoising, variational mode decomposition

## Abstract

Pulsars, especially X-ray pulsars detectable for small-size detectors, are highly accurate natural clocks suggesting potential applications such as interplanetary navigation control. Due to various complex cosmic background noise, the original pulsar signals, namely photon sequences, observed by detectors have low signal-to-noise ratios (SNRs) that obstruct the practical uses. This paper presents the pulsar denoising strategy developed based on the variational mode decomposition (VMD) approach. It is actually the initial work of our interplanetary navigation control research. The original pulsar signals are decomposed into intrinsic mode functions (IMFs) via VMD, by which the Gaussian noise contaminating the pulsar signals can be attenuated because of the filtering effect during signal decomposition and reconstruction. Comparison experiments based on both simulation and HEASARC-archived X-ray pulsar signals are carried out to validate the effectiveness of the proposed pulsar denoising strategy.

## 1. Introduction

**Figure 1:**
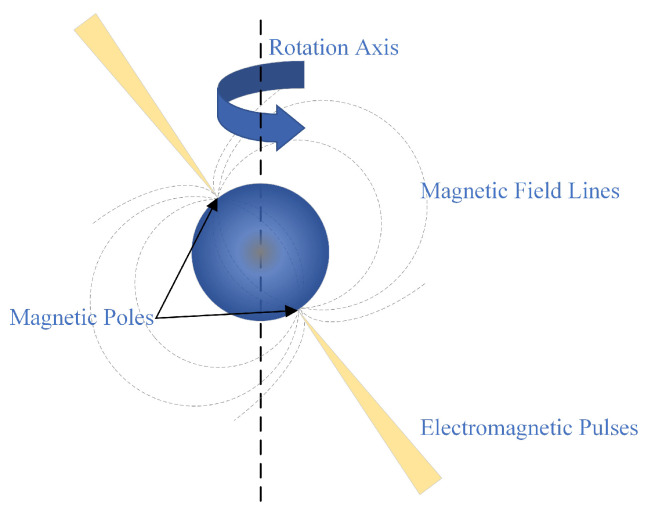
Illustration of the pulsar model.

Pulsars are rapidly rotating neutron stars that emit electromagnetic signals and have periods ranging from milliseconds to thousands of seconds [1,2]; see Figure 1 for an illustration.

The pulsar emission mechanism is complex, featuring chaotic characteristics [3,4]. Various methods have been developed to calculate the Lyapunov exponent and its complementary measures to characterize the time scales on which chaotic systems, such as pulsars, become unpredictable [5,6,7]. Luckily, the potential applications of pulsars, especially accurate pulsar timing features, do not rely on a comprehensive understanding of pulsar emission models. Researchers have found that the rotation periods of pulsars over long timescales are as precise as the state-of-art terrestrial atomic clock, which suggests pulsars are the perfect choice for precise timing and autonomous interplanetary navigation [8,9,10,11]. The precise timing properties of pulsars can be used for unmanned out-terrestrial vehicles, for instance, incorporating advanced robust control algorithms, such as those reported in [12,13], with the pulsar TOA sequence as well as the pulsar positioning algorithm. Nowadays, there are already over 2000 known pulsars that build a firm base for governmental and civilian use, as well as space applications not only for the military but also for astronomy exploration [14,15].

Among all types of pulsars, X-ray pulsars appear to be the most favorable ones as they are detectable by small-size detectors [1,8,9,16,17,18,19]. However, the X-ray pulsar radiation, namely the effective signals, would degenerate into photon sequences with very low intensity when it arrives at the terrestrial detector after long-distance space travel. An even more troublesome problem arises due to the complex, noisy photons, up to about nine times that of the effective photons in quantity, including the diffuse X-ray background and the cosmic X-ray background, which inherently contend with the originally observed signal [11]. These facts imply the low signal-to-noise (SNR) ratio of the pulsar signal and, hence, signify the necessity of pulsar denoising research, especially when the observation time is short.

The pulsar denoising refers to filtering the pulsar profile, generally obtained via epoch folding performed on photon sequences, that stores the time of arrivals (TOAs) of photons [1,17,20]. The Fourier filtering in the frequency domain is the original attempt to denoise pulsar profiles, which has already been proven invalid when the signal profile comprises nonsinusoidal components or nongaussian noise [16,18,21]. The modified kernel regression denoising method reported in [22] develops the second-order derivative compensation and reveals that a clear positive relationship between the TOA accuracy and the profile SNR does not exist. In [23], the wavelet analysis method using noise dispersion to determine the threshold value is applied to denoise the pulsar profile via the wavelet inverse transform. The denoising strategy based on biorthogonal lifting wavelet transforms (WT) reported in [17] studied the statistical properties of the X-ray background noise. The core idea of wavelet transform denoising is choosing an appropriate wavelet library and decomposition level in terms of finding a suitable wavelet basis and threshold function [24]. In particular, the wavelet basis plays an essential role in denoising performance as it is the base of wavelet denoising. However, at present, there is no universal wavelet basis that accommodates different pulsar signals. It has also been shown in [21] that the wavelet denoising methodology heavily relies on expertise and experience and is not compatible with an average processor. New approaches to increase the SNR of X-ray pulsar signals can be found in [25] with a machine learning method and in [26] with a recurrent neural network method, respectively. These two kinds of denoising strategies actually require high computational costs too.

Recently, empirical mode decomposition (EMD) has achieved great success in removing both white and fractional Gaussian noise by reconstructing the signal with pre-determined thresholding intrinsic mode functions (IMFs) [27]. Nevertheless, the traditional EMD framework only allows for analyzing the single signal due to possible mixing phenomena when analyzing multiple signals simultaneously. This problem can be overcome by the multivariate strategy reported in [28], in which multiple pulsar signals are processed at the time while the mode mixing is avoided. The ensemble empirical mode decomposition (EEMD) synthesizes white Gaussian noise in the input signal for the later decomposition, in which the average operation can also avoid the mode mixing problem that existed in the output IMFs after EMD [29,30,31]. Given the limitation from the fixed threshold functions, the adaptive threshold mechanism together with the EMD denoising method shown in [32] remarkably increases the SNR of X-ray pulsar profiles. Via creating the Hausdorff measures between the probability density functions of the input signal and the mode, the filtering approach in [9] aliases and reconstructs the X-ray pulsar profiles to achieve the purpose of denoising. Though the EMD framework allows for analyzing nonstationary and nonlinear signals, it lacks a firm mathematical foundation and is sensitive to sampling noise, which as a particular result, would lead to mode mixing when it is applied on pulsar denoising processing. Additionally, one might find that eliminating the mode mixing problem would build a firm base for performing autonomous mode decomposition and signal denoising, which is essential for embedding advanced control algorithms, such as in [33], into the pulsar-based spacecraft navigation system. Regarding the possible deficiencies of EMD on pulsar signal denoising, Dragomiretskiy developed a novel signal decomposition technique called variational mode decomposition (VMD) that nonrecursively decompose the signal into several quasi-orthogonal IMFs [34]. The VMD has already been applied in many areas such as seismic wave analysis [35], pipeline leakage detection [36] and vibration estimation of rotor-stator machinery [37]. For VMD-based applications, it is important to determine an accurate number of modes that have an essential effect on denoising performance [38]. The detrended fluctuation analysis (DFA) appears to be a favorable choice for this issue as it is a systematic scaling analysis approach to evaluate long-term dependency for nonstationary signal series [35,36]. In our previous work [39], a brief VMD-based denoising algorithm was developed for an X-ray pulsar profile after epoch folding without applying DFA to determine the mode number. To the authors’ best knowledge, no literature reported had shown a detailed discussion about applying the VMD framework on denoising X-ray pulsar signals.

Motivated by the discussion above, this brief undertakes further endeavors on the research of denoising designs of X-ray pulsar signals so as to increase the SNR of the pulsar profiles and pave the way for potential applications. More precisely, a VMD-based denoising strategy is developed. We first perform the epoch folding method on the faint X-ray pulsar signals and obtain the noisy pulsar profile, followed by applying DFA to obtain the number of IMFs, after which the reconstruction of the pulsar signals achieves the denoising goal.

The contribution of this paper includes presenting the denoising algorithm for X-ray pulsar signals based on the VMD method. Compared with those in [18,20,40], the prior knowledge of pulsar profiles is unnecessary for the proposed VMD-based pulsar denoising strategies. In comparison with wavelet analysis [17,23,24], the denoising algorithm in this paper removes the reliance on choosing perfect basis and threshold functions. The VMD-based design in this paper also allows for processing the original pulsar signals that contain nonstationary background noise derived from many sources without leading to mode mixing problems, like that of the EMD-based analysis in [9,28,32].

The rest of the paper is organized as follows. Section 2 introduces basic knowledge of the VMD framework. Section 3 involves the VMD-based denoising design for the contaminated faint X-ray pulsar signals. Section 4 presents the comparison experiments with simulation and measured data. Section 5 concludes this work briefly.

## 2. Preliminaries

### Variational Mode Decomposition

Variational Mode Decomposition (VMD) is a novel framework for signal processing that outperforms the traditional decomposition approaches, allowing for a systematic analysis of nonstationary and nonlinear signals [34]. It is built upon classical theories such as the Wiener filter, Hilbert transformation, frequency mixing, and decomposition method. Compared with the traditional empirical mode decomposition (EMD) technique, the VMD theoretically eliminates the potential problem of mode mixing by decomposing the signal into a sum of IMFs with the limited center frequency and bandwidth calculated analytically. It is worth noting that the reconstruction of the VMD-based decomposed signals, namely summing the obtained IMFs, would attenuate noise and increase SNR.

The VMD-based decomposition performed on the input signal *f* intends to achieve

(1) $$\min_{\left\{h_{k}\right\},\left\{\omega_{k}\right\}}\left\{\sum_{k=1}^{K}{∥\frac{\partial }{\partial t}\left[\left(\delta \left(t\right)+j\frac{1}{\pi t}\right)*h_{k}\left(t\right)\right]e^{-j\omega t}∥}_{2}^{2}\right\},$$

so that the *f* can be reconstructed by the sum $\sum_{k=1}^{K}h_{k}=f$, where $\left\{h_{k}\right\}$ and $\left\{\omega_{k}\right\}$ denote the sets of all modes and center frequencies, respectively; δ(t) stands for Dirac function and notation * denotes convolution. For definitions and discussions about the mode, refer to [34,41]. For the sake of completeness and simplicity, the VMD algorithm renders the constraint problem (1) into an unconstrained one via introducing a quadratic penalty term α and Lagrangian multipliers λ(t), resulting in the following augmented Lagrangian.

(2) $$\begin{array}{cc} L(\left\{h_{k}\right\},\left\{\omega_{k}\right\},\lambda ) & =\alpha \sum_{k=1}^{K}{∥\frac{\partial }{\partial t}\left[\left(\delta \left(t\right)+\frac{j}{\pi t}\right)*h_{k}\left(t\right)\right]e^{-j\omega_{k}t}∥}_{2}^{2}\\ \; & \;+{∥f\left(t\right)-\sum_{k=1}^{K}h_{k}\left(t\right)∥}_{2}^{2}\\ \; & \;+\langle \lambda \left(t\right),f\left(t\right)-\sum_{k=1}^{K}h_{k}\left(t\right)\rangle . \end{array}$$

It then converts the original minimization problem (1) into the saddle point problem of (2). Iterative sub-optimizations in terms of the alternate direction multiplier method (ADMM) can be used to obtain the saddle point of (2) and the optimal solution of (1). Let $\omega_{k}$ and $h_{i\neq k}$ denote the most recently updated values, and the minimization problem for $h_{k}^{n+1}$ becomes

(3) $$\begin{array}{cc} h_{k}^{n+1} & =\underset{h_{k}\in X}{\arg\min}\{\alpha {∥\frac{\partial }{\partial t}\left[\left(\delta \left(t\right)+\frac{j}{\pi t}\right)*h_{k}\left(t\right)\right]e^{-j\omega_{k}t}∥}_{2}^{2}\\ \; & \;+{∥f\left(t\right)-\sum_{t}h_{i}\left(t\right)+\frac{\lambda \left(t\right)}{2}∥}_{2}^{2}\}. \end{array}$$

By applying the Parseval/Plancherel formula for Fourier transform together with Hermitian properties under the $L_{2}$ norm, we can find the optimal solution in the spectrum domain as follows,

(4) $${\hat{h}}_{k}^{n+1}\left(\omega \right)=\frac{\hat{f}\left(\omega \right)-\sum_{i\neq k}{\hat{h}}_{i}\left(\omega \right)+\frac{\hat{\lambda }\left(\omega \right)}{2}}{1+2\alpha {\left(\omega -\omega_{k}\right)}^{2}}.$$

Implementing the same technical principles above, we obtain the center frequency in the form of

(5) $$\omega_{k}^{n+1}=\frac{\int_{0}^{\infty }\omega {\left|{\hat{h}}_{k}\left(\omega \right)\right|}^{2}d\omega }{\int_{0}^{\infty }{\left|{\hat{h}}_{k}\left(\omega \right)\right|}^{2}d\omega }.$$

The specific calculation steps of variational mode decomposition, intuitively including denoising functionality, can be summarized as follows.

1. Initialization: $h_{k}^{1},{\hat{\omega }}_{k}^{1},{\hat{\lambda }}^{1},n\leftarrow 0$;
2. n←n+1;
3. Update ${\hat{h}}_{k}$ and $\omega_{k}$ via (4) and (5), respectively, where k=1,2,…,k,∀ω≥0;
4. Use ${\hat{\lambda }}^{n+1}\left(\omega \right)={\hat{\lambda }}^{n}\left(\omega \right)+\tau \left[\hat{f}\left(\omega \right)-\sum_{k}{\hat{h}}_{k}^{n+1}\left(\omega \right)\right]$ and update $\hat{\lambda }$, ∀ω≥0;
5. Stop the iteration until $\sum_{k=1}^{K}\frac{{∥{\hat{h}}_{k}^{n+1}-{\hat{h}}_{k}^{n}∥}_{2}^{2}}{∥{\hat{h}}_{k}^{n}∥}<\varepsilon$ for a chosen criterion ε, otherwise return to step 2.

Actually, one should calculate an appropriate number of mode *K* before applying VMD to decompose the input signals. For this sake, the DFA approach is applicable to determine *K* as it can characterize different components contained in the signal and provides us with a threshold functionality for the calculation of *K* [21,35,36].

**Remark** **1.** *Adopting the same simulation conditions as our previous work [42], we perform here a comparison experiment of EMD and VMD on decomposing the analog signal defined by*

(6) $$f\left(t\right)=\left\{\begin{array}{c} \cos\left(20\pi t\right),\forall t\in \left[0,0.5\right],\\ \cos\left(20\pi t\right)+\cos\left(6\pi t\right)+\cos\left(120\pi t\right),\\ \;\;\;\;\;\;\;\;\;\;\;\;\forall t\in \left(0.5,0.8\right],\\ \cos\left(20\pi t\right),\forall t\in \left(0.8,1\right]. \end{array}\right.$$

**Figure 2:**
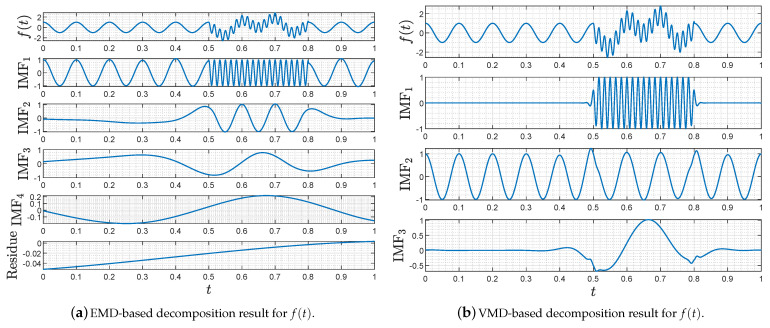
Decomposing results of f(t) via EMD and VMD.

*The simulation results are drawn in Figure 2. As shown in Figure 2a, the EMD decomposes f(t) into four intrinsic mode functions and residue, leading to background signals mixing with frequencies 10 and 60 Hz. This mixing phenomenon distorts the intrinsic mode functions obtained later, lowering the signal decomposition performance. Comparing the results in Figure 2b with that in Figure 2a, the VMD features its superiority by accurately decomposing f(t) into three types of signals with different time frames and frequencies.*

## 3. VMD-Based Denoising Design for X-ray Pulsar Signals

The X-ray pulsar signal observed by detectors contains the time of arrival and the number of photons. It is contaminated by various types of electromagnetic noise. Denoising X-ray pulsar signals appear essential for characterizing different pulsars and building a base for accurate timing in space. In this section, we present a VMD-based denoising method of X-ray pulsars step by step. First, we apply the epoch folding to obtain the noisy pulsar profile. Second, we use the DFA approach to calculate the number of modes of the pulsar profile. Third, we apply the VMD method to decompose and reconstruct the pulsar profile, which achieves the purpose of denoising.

### 3.1. X-ray Pulsar Profile

Epoch folding is applied in this subsection to obtain the pulsar profile. Sort the TOAs of photons into,

(7) $$t_{0}^{\prime }\leq t_{1}^{\prime }\leq t_{2}^{\prime }\leq \ldots \leq t_{N-1}^{\prime },$$

where $t_{i}^{\prime },i\in Z$ denotes the time of arrival of the i+1-th photon. In (7), the symbol “≤” suggests that numerous photons would run into the detector area simultaneously at a certain observing window.

For eliminating the effects of earth-observatory motions and interstellar medium during the pulsar signal transmitting path, we convert the TOAs of photons from observed time $t_{\mathrm{obs}}$ to the solar system barycenter (SSB) as follows,

(8) $$t_{\mathrm{SSB}}=t_{\mathrm{obs}}+\Delta_{E}+\Delta_{R}+\Delta_{S},$$

where $\Delta_{E}$ denotes Einstein delay, $\Delta_{R}$ is the Roemer delay and $\Delta_{S}$ represents the Shapiro delay [43]. Many tools can be applied to finish the time conversion (8). For example, one can synthesis $t_{\mathrm{obs}}$ with the orbit parameters and evoke the barycorr function provided by High Energy Astrophysics Science Archive Research Center (HEASARC) to complete the time conversion automatically.

Denote the TOA sequences in SSB by

(9) $$t_{0}\leq t_{1}\leq t_{2}\leq \ldots \leq t_{N-1},$$

Supposing that the pulsar period is $T_{0}$, the phase of (9) in the normalized time frame [0,1) with respect to the initial time instant can be calculated as

(10) $$\varphi_{i}=\frac{t_{i}-t_{0}}{T_{0}}\mathrm{mod}\;1.$$

Let us averagely scatter each period into *m* bins and compute the number of photons $N_{i}$ in each bin. Construct

(11) $$\chi^{2}=\sum_{i=1}^{m}\frac{{\left(N_{i}-\bar{N}\right)}^{2}}{\bar{N}},\bar{N}=\frac{N}{m}.$$

where *N* is the total number of photons, $\bar{N}$ stands for the mean value of each bin. When we only perform period estimation, the variable $\chi^{2}$ in (11) satisfies the $\chi^{2}$-distribution with m−1 degrees of freedom. Different pulsar periods vary from phases and photon quantities in each bin, which, as a result, shows that the obtained $\chi^{2}$-distributions are different. A period with errors would make the estimated phases inaccurate, simultaneously reducing $N_{i}$, $\bar{N}$ and $\chi^{2}$. In contrast with that, an accurate estimated period suggests that the estimated phases are reliable, and $N_{i}$ and $\bar{N}$ will differ a lot from each other, which increases $\chi^{2}$. Therefore, achieving the best estimation of period *T* relates to finding the maximum of $\chi^{2}$. Let

(12) $$T=\arg_{T\in [T_{\min},T_{\max}]}\max\left\{{Ø}^{2}\right\}.$$

The estimated phase of the arrival time of every photon is assigned to a certain bin according to the pulsar period, resulting in the estimated pulsar profile. The relationship between the phase and the number of photons in each bin is drawn. For instance, the horizontal axis of the pulsar profile is the phase with multiple bins, while the vertical axis denotes the number of photons. Theoretically speaking, the longer the observing time frame and the more the photon quantity, the more accurate the pulsar profile would become [17,20].

### 3.2. Denoise of Pulse Profile Based on VMD

Without causing any confusion, let *f*, defined below, be the pulse profile,

(13) f=x+d,

where *x* is the original pulsar signal, and *d* denotes noise.

The number of modes *K* should be determined before performing VMD on *f*. The *K* plays an essential role in VMD decomposition, or in the view of this brief, the wrong choice of *K* would lower the denoising performance. For these considerations, we apply the detrended fluctuation analysis (DFA) method to determine *K*. The DFA is a favorable scaling tool generally utilized to analyze nonstationary signals, by which the scaling exponent α depicts how tough the signal performs, and a large α means that the volatility is small [21]. The *K* can then be calculated according to α.

Given any signal {u(i),i=1,2,…,N}, let us apply DFA to calculate α by the steps below.

1. Calculate the sum
  (14) $$y\left(k\right)=\sum_{i=1}^{k}u\left(i\right)-k\bar{u},k=1,2,\ldots ,N,$$
  where $\bar{u}=\frac{1}{N}\sum_{i=1}^{N}u\left(i\right)$.
2. Divide the sequence y(k) into $N_{n}=\left(N/n\right)$ nonoverlap length-of-*n* pieces. As for each local trend, one can apply *l*-order polynomial to fit $y_{n}\left(k\right)$. For example, let l=2 and define
  (15) $$y_{n}\left(k\right)=a_{n}k^{2}+b_{n}k+c_{n},$$
  where $a_{n},b_{n}$ and $c_{n}$ denote constants.
3. Define the root-mean-square (RMS) function by
  (16) $$F\left(n\right)=\sqrt{\frac{1}{N}\sum_{k=1}^{N}{\left[y\left(k\right)-y_{n}\left(k\right)\right]}^{2}}.$$
4. Finally, calculate the scaling exponent α by the least square regression approach as follows,
  (17) ln(F(n))=αln(n)+C.
  The relationship between *K* and α can be understood in the sense that the quantity of all scaling exponents $\alpha_{1:K}$ under the constraint $\alpha_{1:K}\geq \theta$ equals *J*, where $\theta =\alpha_{\theta }=0.25$ denotes the threshold and $\alpha_{\theta }=0.5$ for the white Gaussian noise. Without losing generality, we suppose that the noise of the pulsar signal received by probes satisfies the Gaussian distribution.

The variable *J* is determined by $\alpha_{\theta }$ via,

(18) $$J=\left\{\begin{array}{ccc} 1, & \; & \alpha_{0}\leq 0.8\\ 2, & 0.8< & \alpha_{0}\leq 1.0\\ 3, & 1.0< & \alpha_{0}\leq 1.5\\ 4, & \; & \alpha_{0}>1.5 \end{array}\right.$$

Then, we utilize the obtained *K* to decompose the signal *f* via VMD, after which we reconstruct the nominal functions and achieve denoising; see (19).

(19) $$\hat{x}=\sum_{k}h_{k},k=\left\{k|\alpha_{k}\geq \theta \right\}.$$

**Figure 3:**
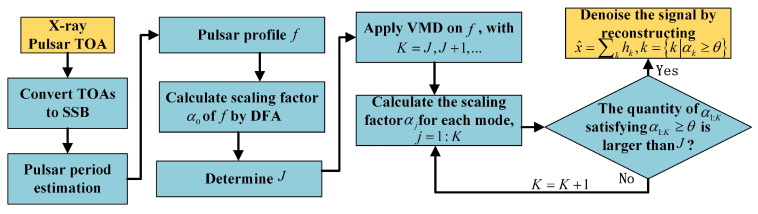
The denoising process of VMD on X-ray pulsar signals.

The flow chart in Figure 3 depicts the VMD denoising process on X-ray pulsar signals. The VMD denoising applied on pulsar signals consists of reconstructing the pulsar profile via summing intrinsic mode functions after decomposition. This process actually attenuates the noise and would remarkably increase the SNR of the pulsar profile. As can be seen, the presented X-ray pulsar denoising strategy does not require any prior knowledge, such as the known pulsar profiles required by the denoising algorithms in [18,20,40].

**Remark** **2.** *The VMD approach provides a general framework for signal processing. In addition to X-ray pulsar denoising designs, various VMD-based applications, such as seismic wave analysis [35], pipeline leakage testing [36] and machinery vibration research [37], have achieved significant attention.*

## 4. Experimental Analysis

This section presents the experimental study focusing on validating the effectiveness of the proposed X-ray pulsar denoising strategies. Both simulated and measured data of the X-ray pulsar are considered. For performance evaluation purposes, various measures, including the Signal to Noise Ratio (SNR), Root-Mean-Square Error (RMSE), and Pearson’s correlation coefficient (PCC), are considered as follows.

$$\begin{array}{cc} \mathrm{SNR} & =10\log\frac{\sum_{i=1}^{M}x{\left(i\right)}^{2}}{\sum_{i=1}^{M}{\left[x\left(i\right)-\hat{x}\left(i\right)\right]}^{2}},\mathrm{RMSE}=\sqrt{\frac{1}{M}\sum_{i=1}^{M}\frac{{\left[x\left(i\right)-\hat{x}\left(i\right)\right]}^{2}}{x{\left(i\right)}^{2}}},\\ \mathrm{PCC} & =\frac{M\sum_{i=1}^{M}x\left(i\right)\hat{x}\left(i\right)-\sum_{i=1}^{M}x\left(i\right)\sum_{i=1}^{M}\hat{x}\left(i\right)}{\sqrt{M\sum_{i=1}^{M}x{\left(i\right)}^{2}-{\left(\sum_{i=1}^{M}x\left(i\right)\right)}^{2}}\sqrt{M\sum_{i=1}^{M}\hat{x}{\left(i\right)}^{2}-{\left(\sum_{i=1}^{M}\hat{x}\left(i\right)\right)}^{2}}}, \end{array}$$

where x(i) denotes the nominal signal, $\hat{x}\left(i\right)$ represents the estimated denoised signal, and *M* stands for the signal length. A higher SNR value or a lower RMSE value leads to better denoising performance. The PCC in the range [−1,1] is generally utilized to address the linear relativity of paired variables. The larger PCC would suggest better denoising performance.

### 4.1. Experiments of Simulation Data

**Figure 4:**
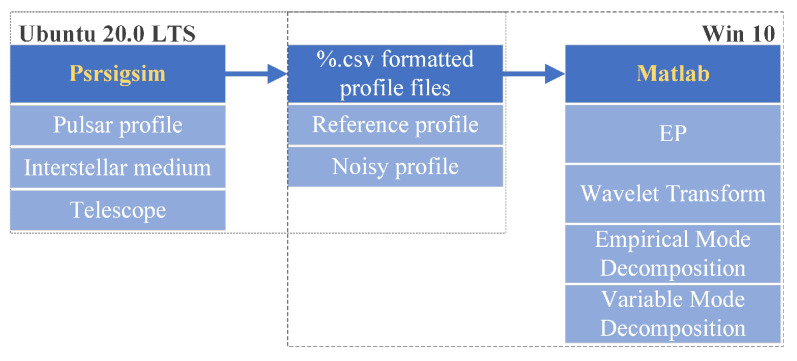
Experiment flow chart of Psrsigsim-propagated pulsar data.

For pulsar simulation purposes, we applied the Psrsigsim pulsar signal simulator to construct a virtual reference X-ray pulsar profile and associated noisy pulsar profile. The Psrsigsim is a python toolbox developed for simulating pulsar signals based on the known and open databases. Parameters including pulsar profile type, interstellar medium, and telescope information should be set before propagating the virtual pulsar signals. In our settings, we saved the pulsar profile data in the portable ‘%.csv’ form and processed the data via Matlab. Figure 4 depicts the overall production of propagating data and processing.

**Figure 5:**
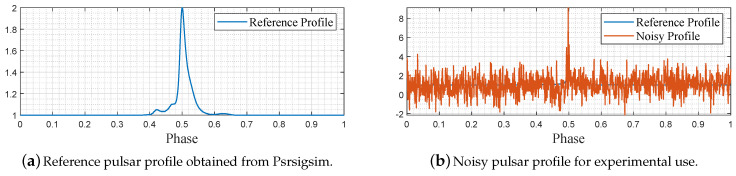
The reference and noisy pulsar profile of simulation data.

We adopted the default pulsar profile ‘J1713 + 07474’ provided by Psrsigsim as the reference profile, by which the noisy pulsar profile is constructed via the ‘pulsar.make_pulse’ function of the Psrsigsim class with a 2000-s observing time. As a result, two million pulsar events in terms of phases were obtained and taken as noisy pulsar signals. The reference pulsar profile is plotted in Figure 5a, while the noisy profiles in the first 1000 samples are drawn in Figure 5b. The vertical coordinate unit is omitted for the convenient processing of simulation data. Two hundred thousand noisy samples were adopted to perform filtering techniques that include epoch folding (EP), wavelet transform (WT), empirical mode decomposition (EMD), and variational mode decomposition (VMD). It is worth noting that the pulsar profile data after EP builds the base for the latter three approaches, or say, the latter three ones use the folding profile as the input source to perform filtering.

**Figure 6:**
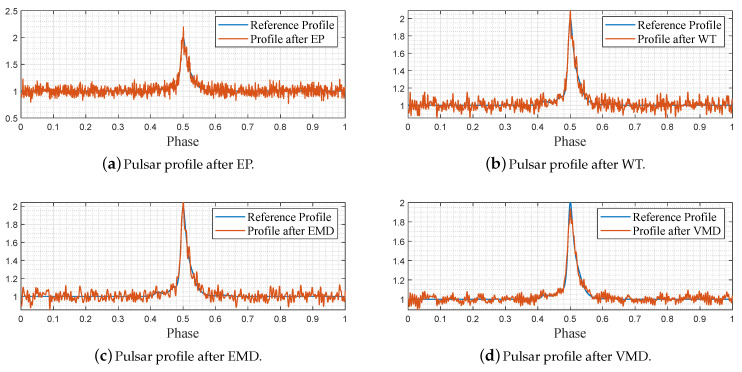
Denoising results of simulation data via different methods.

For EP filtering, we divided the adopted 200,000 noisy data into 1000 bins per the whole period, following the routine of (7)–(11), and depicted the folding results in Figure 6a. For WT filtering, the Matlab widenoise function was applied to denoise the pulsar profile after EP, and we decomposed the profile signal into three layers, after which the coefficients with high frequencies were eliminated manually. The reconstructed filtered profile after WT is shown in Figure 6b. For EMD filtering, the Matlab emd function was directly applied to process the EP-folded profile data sequence, and eight IMFs were obtained in which the last seven ones were summed together to reconstruct the profile signal, as plotted in Figure 6c. Finally, we implemented the proposed VMD-based denoising strategy to denoise the EP-folded profile data and plotted the results in Figure 6d. For VMD decomposition, we determined the mode number K=3 via the DFA, as shown in (13)–(19), after which the VMD function shown in [34] is evoked.

As shown in Figure 5b, the noisy pulsar profile lacks basic profile shape features, which are important to characterize the pulsar property. Figure 6a–d demonstrates the denoising performance of the EP, WT, EMD, and VMD, respectively. Compared with the other three methods, it can be found that our VMD-based denoising strategy obtains better denoising results, as the denoised profile features a more fluent shape and fewer disturbances. For supporting this viewpoint, the SNRs, RMSEs, and PCCs of the original noisy profile and the denoised profile samples are shown in Table 1.

In Table 1, the term ‘ORI’ refers to the original noisy profile samples. As can be seen, all denoising methods chosen for comparison dramatically increase the SNR values compared with the original profiles of negative SNR, which means that the pulsar signal intensity is less than 1/10 of the noise. In particular, our VMD-based denoising strategy outperforms the compared methods as it gained the highest SNR, smallest RMSE, and largest PCC among the others.

### 4.2. Experiments of HEASARC-Archived Data

The real pulsar TOA data are implemented in this subsection to validate the proposed pulsar denoising strategy. We chose the ni1011010301.cl file to perform denoising experiments from the XAMIN database. The chosen ‘%.cl’ file includes the TOAs of Crab Pulsar photons observed and archived by the NICER mission in standard FITS format. The ni1011010301.cl file was created on 20 November 2017 after a 99-min continuous observation of the Crab X-ray Pulsar and includes 1,680,000 observed photons.

**Figure 7:**
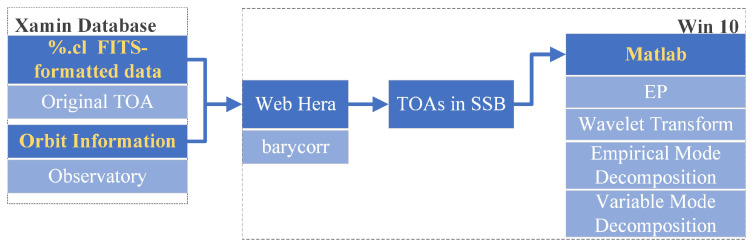
Experiment flow chart of HEASARC-archived pulsar data.

Because the motions of earth and observatory, as well as the interstellar medium, would distort the pulsar signals, we needed to convert the recorded TOAs to the solar system barycenter (SSB). More precisely, the Web Hera, an online HEASARC HEAsoft toolbox set, was applied to evoke the barycorr function that converts the TOA information in ‘%.cl’ files into TOA in SSB, in which the associated orbit information should be set simultaneously. Later on, we performed denoising analysis via EP, WT, EMD, and VMD, respectively. The overall process is depicted in the Figure 7.

**Figure 8:**
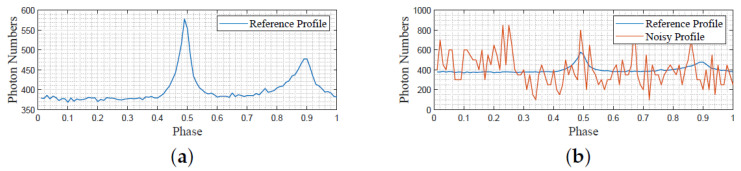
The reference and noisy pulsar profile archived pulsar data. (**a**) Reference pulsar profile obtained via folding all photons; (**b**) Noisy pulsar profile.

According to (12), we estimated the pulsar period by the Chi-square period estimation method. The estimated pulsar period is 0.033742389697 s. As the SNR value becomes large due to an increase in time frames used for folding, we view the pulse profile created by all 1,680,000 observed data as the reference signal while viewing the pulse profile created by the former 40,000 photons as a disturbance signal for experimental use. The reference profile is then obtained by multiplying the nominal signal by the ratio of noisy photon quantity and all photons, in which the phase part is divided into 100 bins. The reference and noisy profiles are depicted in Figure 8a,b.

**Figure 9:**
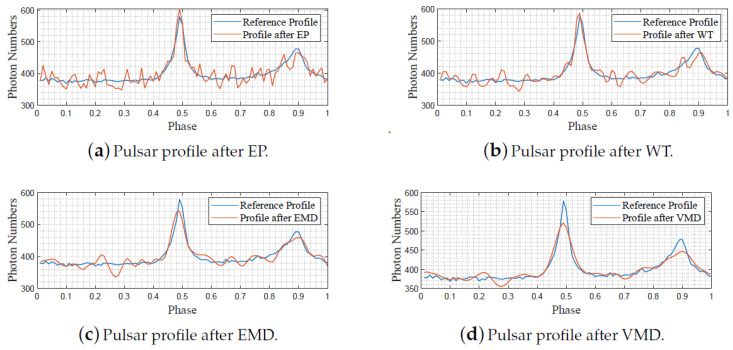
Denoising results of archived pulsar data via different methods.

The EP method was firstly used to obtain the X-ray pulsar profile, and the results are shown in Figure 9a. Note that the obtained profile in Figure 9a includes 400 times of folding because the total photon quantity in use is 40,000, and each period is divided into 100 bins. For WT filtering, we followed the same routine as the previous subsection and manually removed the high-frequency part, and the results are depicted in Figure 9b. For EMD filtering, the EP-folded profile data were decomposed into six modes, and we summed the later five ones to reconstruct the profile. The EMD-based denoising result is shown in Figure 9c. For VMD filtering, we applied the method of (13)–(18) to calculate the number of modes and obtain K=3, based on which we performed VMD decomposition and signal reconstruction. The VMD-based denoising results are shown in Figure 9d.

It is observed from Figure 9a–d that the adopted approaches achieve denoising at different levels. Figure 9c depicts the results after EP, in which obvious saw signals exist, even though the pulse shape matches that of the reference. It is hard to distinguish the best denoising algorithm among WT, EMD, and VMD from the plotted figures. We then numerically summarized the SNRs, RMSEs, and PCCs of the denoised signals in the following table for better comparisons.

The performance indexes in Table 2 indicate that all chosen experimental approaches have realized denoising purposes, and the SNRs are obviously increased compared with the original noisy signal. In particular, the proposed VMD-based denoising algorithm outperforms the other three approaches when inputting virtual Psrsigsim-propagated pulsar data. The obtained experimental results validate the effectiveness of the proposed VMD-based X-ray pulsar denoising design.

## 5. Conclusions

This paper has made further efforts on X-ray pulsar denoising techniques. The VMD approach incorporated with EP and DFA is facilitated to derive a novel pulsar denoising algorithm that features filtering capacity while eliminating the mode mixing problem after EMD decomposition. Experimental results with both simulation and HEASARC-archived pulsar data demonstrate that the proposed denoising algorithm outperforms the EP-, WT- and EMD-based denoising approaches. In the future, the authors will apply the proposed denoising algorithm on pulsar identification and pulsar-based navigation simulation of orbital satellites.

## Figures and Tables

**Table 1:** Denoising performance with simulation data.

Method	ORI	EP	WT	EMD	VMD
SNR	−0.1145	22.8210	25.9410	27.2745	**29.8821**
RMSE	1.0121	0.0727	0.0508	0.0435	**0.0313**
PCC	0.1554	0.8619	0.9251	0.9441	**0.9668**

**Table 2:** Denoising performance with measured data.

Method	Ori	EP	WT	EMD	VMD
SNR	7.8000	25.9117	27.9890	29.1157	**30.3373**
RMSE	0.4132	0.0506	0.0391	0.0339	**0.0270**
PCC	0.0462	0.8748	0.9138	0.9277	**0.9480**

## Data Availability

The HEASARC-archived %.cl file is available at https://heasarc.gsfc.nasa.gov (accessed on 20 November 2017).
